# ANATOMICAL VARIATIONS OF PORTAL VENOUS SYSTEM: IMPORTANCE IN SURGICAL CLINIC

**DOI:** 10.1590/0102-672020210002e1666

**Published:** 2022-06-24

**Authors:** Edmundo Vieira PRADO, Andy PETROIANU

**Affiliations:** 1 Hospital das Clínicas da Faculdade de Medicina de Marília, Departamento de Cirurgia do Aparelho Digestivo, Marília - SP - Brazil;; 2 Faculdade de Medicina da Universidade Federal de Minas Gerais, Departamento de Cirurgia, Belo Horizonte - MG - Brazil.

**Keywords:** Anatomy, Portal System, Portal Vein, Mesenteric Veins, Splenic Vein, Anatomia, Sistema Porta, Veia Porta, Veias Mesentéricas

## Abstract

Knowledge of the portal system and its anatomical variations aids to prevent surgical adverse events. The portal vein is usually made by the confluence of the superior mesenteric and splenic veins, together with their main tributaries, the inferior mesenteric, left gastric, and pancreaticoduodenal veins; however, anatomical variations are frequent.

**AIM:**

This article presents a literature review regarding previously described anatomical variations of the portal venous system and their frequency.

**METHODS:**

A systematic review of primary studies was performed in the databases PubMed, SciELO, BIREME, LILACS, Embase, *ScienceDirect,* and Scopus. Databases were searched for the following key terms: *Anatomy, Portal vein, Mesenteric vein, Formation, Variation, Variant anatomic, Splenomesenteric vein, Splenic vein tributaries,* and *Confluence*.

**RESULTS:**

We identified 12 variants of the portal venous bed, representing different unions of the splenic vein, superior mesenteric vein, and inferior mesenteric vein. Thomson classification of the end of 19th century refers to the three most frequent variants, with type I as predominant (M=47%), followed by type III (M=27.8%) and type II (M=18.6%).

**CONCLUSION:**

Thomson classification of variants is the most well-known, accounting for over 90% of portal venous variant found in clinical practice, inasmuch as the sum of the three junctions are found in over 93% of the patients. Even though rarer and accounting for less than 7% of variants, the other nine reported variations will occasionally be found during many abdominal operations.

## RESUMO - RACIONAL

O conhecimento do sistema porta e de suas variações anatômicas contribui para prevenir acidentes cirúrgicos. Usualmente, a veia porta é formada pela confluência das veias mesentérica superior e esplênica, junto com suas principais tributárias: as veias mesentérica inferior, gástrica esquerda e pancreaticoduodenal. Entretanto, variações anatômicas são frequentes.

**OBJETIVO:**

Este artigo apresenta uma revisão da literatura em relação às variações anatômicas previamente descritas do sistema venoso porta e sua frequência.

**MÉTODOS:**

Foi realizada revisão sistemática de estudos primários nas bases de dados PubMed, Scielo, BIREME, LILACS, Embase, Science Direct e Scopus. As bases de dados foram pesquisadas pelas seguintes palavras-chave: Anatomia, Veia porta, Veia mesentérica, Formação, Variação, Variante anatômica, Veia esplenomesentérica, Veia esplênica, tributárias e Confluência.

**RESULTADOS:**

Foram identificadas doze variantes do sistema venoso portal, representando diferentes formações da veia esplênica, veia mesentérica superior e veia mesentérica inferior. A classificação de Thomson, do final do século XIX, refere três variantes mais frequentes, com predomínio do tipo I (M = 47%), seguido do tipo III (M = 27,8%) e do tipo II (M = 18,6%).

**CONCLUSÃO:**

A classificação de variantes de Thomson é a mais conhecida e responde por mais de 90% da variante venosa portal encontrada na prática clínica, na medida em que a soma das três junções é encontrada em mais de 93% dos pacientes. Embora mais raras e representando menos de 7% das variantes, as outras nove variações relatadas ocasionalmente serão encontradas durante muitas cirurgias abdominais.

## INTRODUCTION

The abdominal portal vein (PV) starts at the level of the second lumbar vertebra, anterior to the inferior vena cava and posterior to the pancreatic neck. It is composed of the hepatic pedicle, posterior to the hepatic artery and to the common bile duct. The PV is formed by the convergence of superior mesenteric vein (SMV) and splenic vein (SV), measures about 6.5 cm in length and 0.8 cm in diameter on average^6^ ^,^ ^9^ ^,^ ^11^. Its main tributaries are the left gastric vein, which ends at its left border; the pancreaticoduodenal vein, superoposteriorly close to the head of the pancreas^9^ ^,^ ^11^; and the veins proceeding from the small and large intestines (SMV and inferior mesenteric vein [IMV]). Other tributaries of the hepatic PV are the cystic veins, proceeding from the gallbladder; pancreatic veins; and right and left gastroepiploic vessels, besides the short gastric veins through the splenic and right gastric veins. The IMV receives blood from the upper part of the rectum, sigmoid, and descending colon^6^ ^,^ ^9^ ^,^ ^11^ ^,^ ^14^. IMV is predominantly ventral and to the left of the superior mesenteric artery, at the level of the third portion of the duodenum together with the duodenojejunal flexure. The SV is formed by 5-15 venules, originated at the red pulp of the splenic parenchyma, which join together close to the tail of the pancreas. Then, the SV receives as tributaries the short gastric veins in variable number; pancreaticoduodenal veins, also variable in number; and posterior gastric veins, including, eventually, the left gastric vein as well as the IMV. It must be emphasized that the splenogastric vessels are independent and are not among the tributaries of the SV, despite that communicating vessels could occur among them. The SV continues in a dorsal sulcus of the pancreas toward the direction of its head, which can be visible through the lower border of the pancreas. The blood supply of the SV comes from the spleen, larger curvature of the stomach, pancreas, left half of the colon, upper rectum, and retroperitoneum^6^.

The SMV is formed by tributaries of the small intestine, right colon, head of the pancreas, and part of the stomach - through the right gastroepiploic vein. Its position is predominantly ventral and is a short vein that is formed from multiple tributaries as they cross the third portion of the duodenum to the right of the duodenojejunal junction, close to the uncinate process^11^.

This presentation of the abdominal portal system is the most commonly found; however, there are variations which are the reason of the study among the anatomists for more than a century ago. The first work relating variations of the PV tributaries was published by Thomson et al., which distributed them into three types^25^:

- Type I - IMV as tributary of the SV
- Type II - trifurcation in the PV, formed by the union of the SMV, IMV, and SV
- Type III - IMV as tributary of the SMV

Despite that there are many anatomical works on the PV, its variations still continue to be described and, sometimes, they are unprecedented. Benninger et al. suggested another tributary of the PV, i.e., the splenomesenteric vein^3^. Recently, it was also described as modification to Thomson classification^14^, with variations of the left gastric vein as direct tributary of the PV or the SV.

The objective of this study was to review the literature related to anatomical variations of the PV system and their frequency, accentuating the morphological knowledge and its surgical applicability, which may aid to prevent surgical adverse events.

## METHODS

**Figure 1 -:**
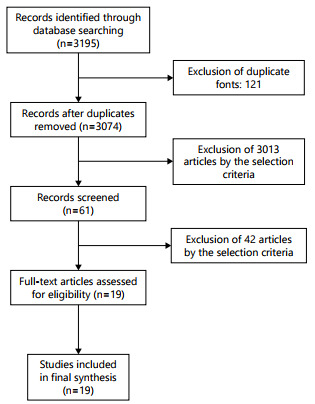
Flow diagram of literature selected articles related to abdominal portal vein system.

Systematic review of primary studies was performed with the elaboration based on the *Checklist Preferred Reporting Items for Systematic Reviews and Meta-Analyses* (PRISMA)^18^ in the databases such as PubMed.gov, SciELO, BIREME, LILACS, Embase, *ScienceDirect,* and Scopus. In the search strategy, the uniterms used were as follows: *Anatomy, Portal vein, Mesenteric vein,* Formation, Variation, Variant anatomic, Splenomesenteric vein, Splenic vein tributaries, and Confluence. The research included MeSH/DeCS/Emtree and Allfiels, excluding animals, *in vitro* studies, studies published in congress annals, secondary studies, and articles with inexplicit method. The included articles were only on humans with studies in cadavers, imaging examination, reports, and case series. Complete articles in English, Spanish, and Portuguese were studied without restriction from the institution of origin nor year of publication (Figure 1).

Only 61 articles were found for reading in their entirety, as well as the tile of the articles of their respective bibliographical references. After reading all texts, 19 were selected for this review, 18 with studies discussing (n=20) and 1 study reporting a case not described yet in the literature with a total of 2418 cases in 11 countries. Only one work investigated the PV during the operative act (Table 1). The other articles were not included in this review because they do not discuss the frequency of the variations, do not present anatomical descriptions of the variations found, and do not present statistical data of measuring the frequency of the variations of the portal system.

**Table 1 -:** Thomson classification of abdominal portal vein system published in selected literature studies in number of cases (N) and (percentages).

Author, year (country)	N	Method	Type I N (%)	Type II N (%)	Type III N (%)	Others (types IV-XII) N (%)
Thomson et al., 1890 (England)	118	Cadaver dissection	71 (60.1)	8 (6.7)	39 (33)	
Walcker et al., 1922 (the United States)	150	Cadaver dissection	69 (46)	33 (22)	48 (32)	
Gilfillan et al., 1950 (the United States)	54	Cadaver dissection	30 (55.6)	8 (14.8)	16 (29.6)	
Purcell et al., 1951 (the United States)	100	Cadaver dissection	28 (28)	3 (3)	53 (53)	16 (16)
Duques et al., 2000 (Brazil)	56	Cadaver dissection	42 (75)	2 (3.6)	12 (21.4)	
Cabrera et al., 2005 (Cuba)	20	Cadaver dissection	15 (75)	2 (10)	3 (15)	
Ibukuro et al., 1996 (Japan)	43	Angiographies	19 (46)	11 (25)	13 (29)	
Graf et al., 1997 (the United States)	51	Angiographies	28 (55)	9 (17)	14 (27)	
Misuta et al., 2004 (Japan)	27	Angiographies	14 (51.8)	4 (14.8)	9 (33)	
Kim et al., 2007 (South Korea)	205	Angiographies	112 (53)	26 (12)	67 (31)	
Zhang et al., 2007 (China)	191	Angiographies	86 (45)	34 (18)	71 (37)	
Gorantla et al., 2007 (India)	01	Cadaver dissection	00	00	00	01
Sakaguchi et al., 2010 (Japan)	87	Angiographies	63 (68.5)	7 (7.6)	17 (18.5)	
Chaijaroonkhanarak et al., 2010 (Thailand)	65	Cadaver dissection	38 (69.1)	10 (15.38)	17 (30.9)	
Krumm et al., 2011 (Germany)	916	Angiographies	344 (37.6)	266 (28.8)	176 (19.2)	130 (14.2)
Benninger et al., 2013 (the United States + Lebanon)	53	Cadaver dissection	38 (71.1)	5 (9.43)	10 (18.9)	
Khamanarong et al., 2015 (Thailand)	211	Cadaver dissection	117 (56.2)	3 (1.4)	91 (43.7)	
Rault and Bahetee, 2015 (India)	40	Cadaver dissection	12 (30)	19 (47.5)	9 (22.5)	
Kaur et al., 2016 (India)	30	Cadaver dissection	15 (50)	3 (10)	12 (40)	
Total	2418		1141 (47.1)	453 (18.7)	677 (27.9)	147 (6.0)

**Figure 2 -:**
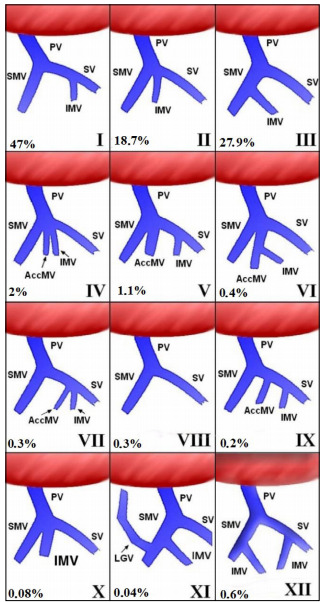
Some anatomical variations of the abdominal portal system structure, according to Thomson (1890) classification ^23^. Type I: IMV as a tributary of the SV; type II: three veins constitute the portal vein, i.e. SMV, IMV, and SV; type III: IMV as a tributary of the SMV; type IV: AccMV at the angle of portal confluence; type V: two SMVs and the IMV constitute the PV; type VI: IMV is tributary of the SMV; type VII: AccMV is tributary of the PV together with the IMV; type VIII: absence of IMV; type IX: AccMV as tributary of the PV; type X: two SMVs constitute the PV; type XI: LGV as tributary of the SMV; type XII: two IMVs, one a tributary of the SMV and the other a tributary of the SV. PV: portal vein, SV: splenic vein, SMV: superior mesenteric vein, IMV: inferior mesenteric vein, AccMV: accessory mesenteric vein, LGV: left gastric vein.

There are 12 variations described relative to PV system through the union of the SV, SMV, and IMV (Figure 2), such that Thomson (1890) classification refers to the three most frequent, with predominance of type I (28-75%, M=47%), followed by type III (15-40%, M=27.8%) and type II (1.4-28.8%, M=18.6%). The three variations described by Thomson were the only ones described in 16 out of 19 articles. The rest are rarer and make a total of 5.2% of the cases^2^ ^,^ ^8^ ^,^ ^9^ ^,^ ^10^ ^,^ ^12^ ^,^ ^13^ ^,^ ^15^ ^,^ ^16^ ^,^ ^17^ ^,^ ^20^ ^,^ ^21^ ^,^ ^22^ ^,^ ^25^ ^,^ ^27^.

Krumm et al. ^16^ presented six variants that were not previously described: type IV (n=70-2%), in which an accessory mesenteric vein (AccMV) enters at the angle of portal confluence as in Thomson type II; type V (n=28-1.1%), similar to Thomson variant I with two equal SMVs and the introduction of the IMV into the PV; type VI (n=10-0.4%), similar to variant V, that is, the IMV enters in one of the SMV; type VII (n=8-0.3%), similar to variant I, in which an AccMV enters into the PV at the IMV introduction site; type VIII (n=8-0.3%), in which the IMV is absent; type IX (n=6-0.2%), similar to Thomson type I, with an AccMV which discharges in the PV between the IMV and the confluence of the SMV and PV; and type X (n=2-0.08%), with two equal mesenteric trunks at the confluence of the PV. Gorantla et al. ^10^ reported type XI, in which the left gastric vein is introduced into the SMV (n=1-0.04%). Purcell et al. ^20^ described 16 cases of type XII, where there were 2 IMVs: a tributary of the SMV and another from SV (0.6%).

According to Krumm et al. ^16^, the variations can be distributed into three IMV introduction groups as follows:

- Group 1: IMV is tributary of SV (variants I, V, VII, and IX).
- Group 2: IMV is tributary of SMV (variants III, VI, and XI).
- Group 3: It does not fit into group 1 or 2 (variants II, IV, VIII, and X).

Variant XII is an exception because there are two IMVs, which can be included in groups 1 and 2.

## DISCUSSION

Thomson variants are the most well known in the surgical practice, inasmuch as the sum of the three junctions are found in over 90% of the patients. Less than 7% of the cases form the set of other nine variations. Even though rare, these variations are possible to be found during operations such as gastroduodenopancreatectomy (Whipple surgery)^1^ ^,^ ^7^ ^,^ ^24^, colectomies^28^, venous bypasses due to portal hypertension (PH)^5^ ^,^ ^19^, hepatectonies^19^ and liver transplants^26^, as well as in diverse operations on the pancreas and extrahepatic biliary pathways.

In minimally invasive surgeries, and, most recently, in those performed with the aid of remotely guided robotic devices, perfect knowledge of the anatomical structures and their variations became indispensable in abdominal operations, mainly those who have visceral venous times, all pertaining to the portal system^28^. Variations in the vascular architecture are the common causes of operative accidents with consequent increasing in surgical time and of the postoperative hospitalization period^27^.

Portal hypertension is one of the diseases with multiple complications, including cirrhosis, schistosomiasis, retroperitoneal and biliopancreatic tumors, as well as adjacent arterial aneurysms and right heart failure^4^ ^,^ ^5^ ^,^ ^19^. With the increase in pressure, the PV system is reorganized, with the increase in caliber of the veins, such as PV (>13 mm), SMV, and SV (>10 mm), associated with splenomegaly^5^ ^,^ ^19^.

The anatomy of the portal system is also important in portal thrombosis (PT)^26^. The disease is classified into four types in accordance with the stricken venous system and clinical manifestations as follows: type I: asymptomatic isolated SV thrombosis; type II: asymptomatic intrahepatic PV thrombosis without PH; type III: asymptomatic diffused PT; and type IV: isolated or diffused symptomatic PT^26^. It occurs, in general, without known cause and is transitory; however, it has been more widely studied after splenectomy. Usually, it is asymptomatic, and there is no drug treatment yet that may prevent it or promote vascular rechanneling. Eventually, its clinical practice is associated with fever PT, abdominal pain, diarrhea, ileodynamic, ascites, and bleeding of esophageal varices^23^ ^,^ ^26^ ^,^ ^29^.

The knowledge of the abdominal portal system anatomy allows for the planning of venous bypasses to alleviate PH, mainly when associated with upper digestive hemorrhage. The bypasses include the right portocaval or with prosthesis, mesentericocaval, centralized and distal splenorenal, and left gastric caval.

The knowledge of portal anatomical variations helps also to understand the hepatofugal blood flow in the cases of PH^1^. More than 20 pathways have been described; for example, the reflux for inferior mesenteric collateral vessels that are connected through the hemorrhoidal plexus^1^.

## CONCLUSION

Thomson classification of variants is the most well-known, accounting for over 90% of PV variant found in clinical practice, inasmuch as the sum of the three junctions are found in over 93% of the patients. Even though rarer and accounting for less than 7% of variants, the other nine reported variations will occasionally be found during many abdominal operations.
